# P-1261. In Vitro Persistence of Clinical Methicillin-Resistant Staphylococcus aureus (MRSA) Isolates from Bone and Joint infections after Exposure to Daptomycin (DAP), Telavancin (TLV), and Vancomycin (VAN)

**DOI:** 10.1093/ofid/ofaf695.1452

**Published:** 2026-01-11

**Authors:** Aliaa Fouad, Joseph L Kuti, Christian M Gill

**Affiliations:** Hartford Hospital, Farmington, CT; Hartford Hospital, Farmington, CT; Hartford Hospital, Farmington, CT

## Abstract

**Background:**

Bacterial persistence after antimicrobial exposure is well described for MRSA and maybe responsible for relapsing infections. However, the impact of antibiotic selection and their role on persistence in recurrent MRSA bone and joint infections (BJIs) requires further investigation. Determining the persister profiles of DAP, TLV, and VAN may inform optimal selection of therapy for MRSA BJIs.

**Table 1.**
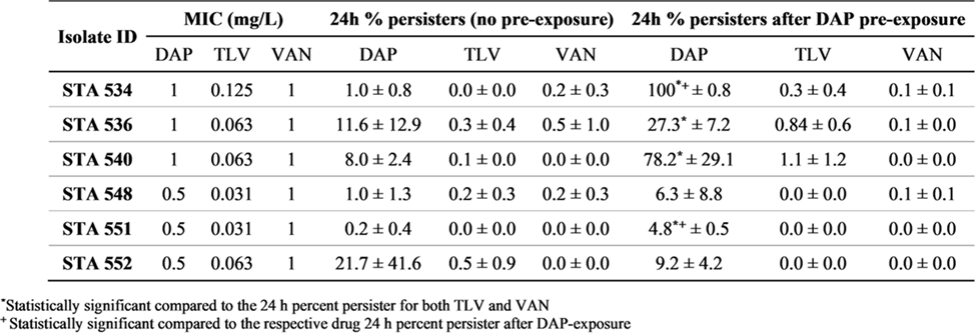
Modal MICs and 24 h percent persisters for DAP, TLV, and VAN to the respective control timepoint against six MRSA isolates with and without DAP pre-exposure. The 24h percent persister values are the mean ± SD of two replicates for each isolate.

**Methods:**

Bacterial persistence was evaluated using quantitative *in vitro* assays for six clinical MRSA isolates from BJIs and one quality control ATCC *S. aureus* strain. All isolates were susceptible to the three test agents per CLSI interpretation. The assays were conducted in duplicates during the mid-exponential growth phase. Isolates were then exposed to antimicrobial concentrations simulating the free-steady-state average concentration for each agent. Persisters were quantitated as the percent of the total population quantifiable at 24 h compared with the respective control. The 24 h percent persister population was also quantified after three serial passages of DAP pre-exposure at concentrations equal to 2 x MIC. Percent persisters were compared using one-way ANOVA and *p*-value < 0.05 was considered statistically significant.

**Results:**

A relatively low percent of persisters were observed at 24 h across all 6 MRSA (< 21.7% for DAP, ≤0.5% for TLV and VAN (Table 1). No significant differences between the three test agents were observed in quantitative persister assays without DAP pre-exposure. After three days of *in vitro* DAP pre-exposure, the 24 h percent persisters significantly increased compared with no pre-exposure experiments for two DAP-treated isolates (STA 534: 100% versus 1%, STA 551: 4.8% versus 0.2%). The 24 h percent persisters were significantly higher for DAP compared with TLV and VAN in four isolates (STA 534, 536, 540, 551). There was no significant increase in the 24 h percent persister for TLV and VAN groups after DAP pre-exposure compared with no pre-exposure.

**Conclusion:**

DAP, TLV, and VAN at clinically achievable concentrations resulted in similar bacterial persistence without pre-exposure. Pre-exposure with DAP did not increase persistence for TLV and VAN. Additional clinical studies are needed to differentiate between these anti-MRSA agents in the treatment of MRSA BJIs.

**Disclosures:**

Joseph L. Kuti, PharmD, Abbvie: Advisor/Consultant|Abbvie: Grant/Research Support|Abbvie: Honoraria Christian M. Gill, PharmD, Cepheid: Grant/Research Support|Cumberland Pharmaceuticals: Grant/Research Support|Entasis: Grant/Research Support|Everest Medicines: Grant/Research Support|Shionogi: Grant/Research Support

